# A Programmable High-Voltage Compliance Neural Stimulator for Deep Brain Stimulation *in Vivo*

**DOI:** 10.3390/s150612700

**Published:** 2015-05-28

**Authors:** Cihun-Siyong Alex Gong, Hsin-Yi Lai, Sy-Han Huang, Yu-Chun Lo, Nicole Lee, Pin-Yuan Chen, Po-Hsun Tu, Chia-Yen Yang, James Chang-Chieh Lin, You-Yin Chen

**Affiliations:** 1 Department of Electrical Engineering, Chang Gung University, No. 259 Wen-Hwa 1st Rd., Guishan Township, Taoyuan County 333, Taiwan; E-Mails: alex.mlead@gmail.com (C.-S.A.G.); scarjei@gmail.com (J.C.-C.L.); 2 Portable Energy System Group, Green Technology Research Center, College of Engineering, Chang Gung University, No. 259 Wen-Hwa 1st Rd., Guishan Township, Taoyuan County 333, Taiwan; 3 Interdisciplinary Institute of Neuroscience and Technology, Zhejiang University, Zhouyiqing Building, Yuquan Campus, Zhejiang University, Hangzhou 310027, China; 4 School of Medicine, Chang Gung University, No. 259 Wen-Hwa 1st Rd., Guishan Township, Taoyuan County 333, Taiwan; 5 Department of Biomedical Engineering, National Yang Ming University, No.155, Sec.2, Linong St., Taipei 112, Taiwan; E-Mail: seanhuang26@gmail.com; 6 Center for Optoelectronic Medicine, National Taiwan University College of Medicine, No.1 Jen Ai Rd. Sec. 1. Taipei 100, Taiwan; E-Mail: yuchunaricalo@gmail.com; 7 Department of Bioengineering, University of California, San Diego, 9500 Gilman Drive #0412, La Jolla, CA 92093, USA; E-Mail: nal006@ucsd.edu; 8 Department of Neurosurgery, Chang Gung University and Memorial Hospital at Linkou, No.5, Fuxing St., Guishan Township, Taoyuan County 333, Taiwan; E-Mails: pinyuanc@cgmh.org.tw (P.-Y.C.); d12096@cgmh.org.tw (P.-H.T); 9 Department of Biomedical Engineering, Ming-Chuan University, 5 De Ming Rd., Guishan Township, Taoyuan County 333, Taiwan; E-Mail: cyyang@mail.mcu.edu.tw

**Keywords:** deep brain stimulation (DBS), complementary metal-oxide-semiconductor (CMOS), high-voltage compliance neural stimulator

## Abstract

Deep brain stimulation (DBS) is one of the most effective therapies for movement and other disorders. The DBS neurosurgical procedure involves the implantation of a DBS device and a battery-operated neurotransmitter, which delivers electrical impulses to treatment targets through implanted electrodes. The DBS modulates the neuronal activities in the brain nucleus for improving physiological responses as long as an electric discharge above the stimulation threshold can be achieved. In an effort to improve the performance of an implanted DBS device, the device size, implementation cost, and power efficiency are among the most important DBS device design aspects. This study aims to present preliminary research results of an efficient stimulator, with emphasis on conversion efficiency. The prototype stimulator features high-voltage compliance, implemented with only a standard semiconductor process, without the use of extra masks in the foundry through our proposed circuit structure. The results of animal experiments, including evaluation of evoked responses induced by thalamic electrical stimuli with our fabricated chip, were shown to demonstrate the proof of concept of our design.

## 1. Introduction

In recent decades, electrical stimulation devices have been increasingly used in clinical applications [1,2,3,4] and late-stage development of many traditional “medical” disorders [5,6,7,8]. In particular, deep brain stimulation (DBS) has become one of the most important therapies in functional neurosurgery. The DBS device can deliver tiny electrical signals to modulate neuronal activation in the brain to improve patients’ symptoms. 

**Figure 1:**
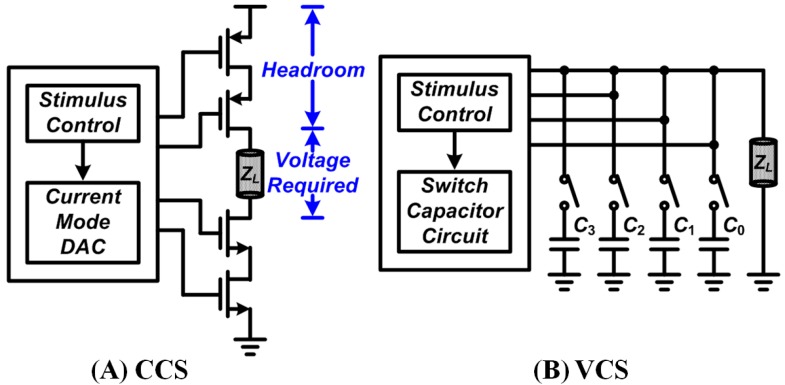
Two main stimulation modes: (**A**) Current-controlled stimulation (CCS) and (**B**) Voltage-controlled stimulation (VCS).

DBS therapy commonly utilizes constant voltage stimulation, with current delivery to the tissue as a function of electrode–tissue impedance (shown in Figure 1). Theoretically, neurons at the targets of interest can be activated or inhibited by an injected “charge” that is generated with the applied voltage or current [9,10,11,12,13,14]. In general, scientific researchers and clinicians can employ an arbitrary waveform with a current/voltage source and/or sink to accumulate certain charge on the cells or tissues by adjusting the pulse widths. The stimulators can consist of monophasic (monopolar) or biphasic (bipolar) configurations according to the purpose of the therapy, where at least two electrodes are required for both of these configurations [9,10,11,12,13,14]. In a biphasic current-mode stimulation, it is usually a leading “cathodic” phase followed by an “anodic” counterpart as a result of the depolarization of the membrane [9,10,11,12,13,14].The first phase is used as stimulation, while the second one fulfills a charge balance to prevent any tissue or cell damage that can arise from accumulated residual charges. Although biphasic stimulation can be utilized for charge balance for the reduction of tissue damage, it has been shown that both phases can contribute to altered neuronal activities [15]. Miller *et al.* reported that biphasic stimulations require higher current than monophasic stimulations for the phase pulse width ranging from 20 to 400 μs, to induce the evoked compound action potential in the animal models such as guinea pig and cat [15,16]. However, despite the difference in charge induction method, the resulting electrophysiological effects of mono- and bi-phasic stimulations should be identical.

A previous study has reported that the use of an arbitrary voltage waveform with identical configuration can achieve successful stimulation with adjustable pulse widths according to the electrode-tissue or electrode-cell impedance [14]. The voltage-mode stimulator features low output impedance with reduced output headroom, where the device is able to drive an almost full voltage swing output with negligible switching loss. However, these conventional voltage-mode stimulators require several weighted capacitors to produce the action potential by controlling the accumulated charge [14]. The weighted capacitors involved in the conventional voltage-mode stimulator are usually large and difficult to realize (~200 μF) in silicon-related semiconductors. Furthermore, the inclusion of these capacitors is not cost-effective and can compromise the ultimate goal of full device integration. 

In contrast, current-controlled stimulation has a theoretical advantage over voltage-controlled stimulation by avoiding variations in the stimulating current caused by fluctuations in brain tissue, which can result in charge interference or impedance changes. However, these current-controlled devices often come with penalties, such as increased output headroom and reduced voltage swing output. These penalties are the result of an increased switching loss caused by high output impedance. Moreover, most existing DBS implants, where most of the technologies were inherited from pacemakers, were controlled by VCS [14]. In short, in terms of conversion efficiency, which is defined as the ratio of effective energy transferred to the load represented by total energy sourced from its power supply to the target of interest, the voltage-mode stimulator outperforms its current-mode counterpart with an almost “full-swing” output. Device energy efficiency is of utmost importance for some applications where the lifetime of the implantable devices should be as long as possible to avoid additional surgeries [17].

The success of DBS in treating movement disorders has led to investigations of its use for psychiatric illnesses such as Tourette’s syndrome, obsessive compulsive disorder, and depression [18]. For the treatment of psychiatric disorders, the DBS used requires high voltage (5 V~10.5 V), frequencies of 100 to 180 Hz, and pulse widths of 90 to 210 ms [19,20,21]. The high-voltage design is required for the delivery of sufficient charge stimulation in DBS. The long-term stability of the electrode–tissue impedance may be required to maintain optimal output driving capability with deep brain implants and to permit appropriate delivery of neuromodulation therapy [22,23]. Although the tissue-electrode impedance in chronic DBS therapy by voltage stimulation is an important parameter for influencing stimulation efficiency, a previous investigation has demonstrated that no significant change in the electrode therapeutic impedance was observed over time [24].

Motivated by the goals of achieving high-voltage implementations with reduced cost and high conversion efficiency, the rest of this study demonstrates the design and evaluation of an efficient high-voltage DBS. The DBS in this study was designed based on the same concept as the conventional voltage-mode stimulator, but without weighted capacitors. The capacitor-less design was demonstrated to be cost-effective for a full integration of system on chip (SoC). It is known that electrical stimulation preferentially activates axons as opposed to cell bodies [25,26,27]. The use of DBS was proposed to produce inhibition of the stimulated area by activation of efferent and afferent axons [25,26]. Thus, a system level implementation of our custom-made DBS platform was performed to investigate the upstream or downstream deep brain nuclei neuronal activation in response to grading DBS stimulations.

## 2. Materials and Methods

### 2.1. Schematic of in Vivo Deep Brain Stimulation (DBS)

**Figure 2:**
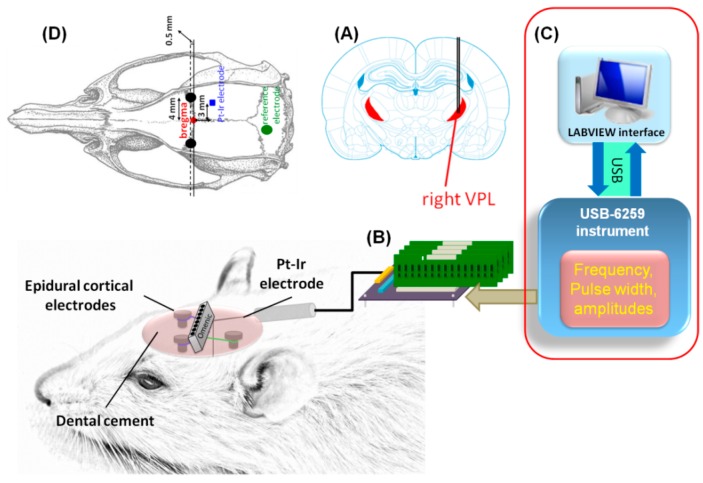
Schematic diagram of *in vivo* DBS experiment. (**A**) The DBS was implemented at the right ventral posteriolateral (VPL) thalamic nuclei in the rat brain; (**B**) The DBS was controlled by a virtual instrument (LabVIEW^®^) with the use of an USB-DAQ equipment; (**C**) The voltage was delivered through a two-channel platinum-iridium electrode; (**D**) Two epidural cortical electrodes (indicated by two black circles) were secured into the skull over the bilateral primary somatosensory cortex of the forepaw (S1FL) for evoked ECoG recordings and a stainless steel screw (indicated by the green circle) was used as the reference electrode.

The conceptual drawing proposed and used for the DBS animal experiments is shown in Figure 2. Our DBS system was connected to a bipolar platinum-iridium electrode (MS303/9-B/SPC, PlasticsOne Inc., Roanoke, VA, USA) implanted at the ventral posterior thalamic complex. 

All stimulus parameters were created using LabVIEW^®^ (ver. 8.6, National Instruments Corp. Austin, TX, USA) and generated as a conventional pulse-width modulation (PWM) scheme through a data acquisition (DAQ) module (NI USB-6259, National Instruments Corp., Austin, TX, USA). The analog stimulus output was channeled into our proposed stimulator. Meanwhile, the electrocorticography (ECoG) recordings were performed with subdural electrodes that were synchronized with the DBS pulse with a repetition rate of 3 Hz.

### 2.2. Design of Stimulator with High-Voltage Compliance

**Figure 3:**
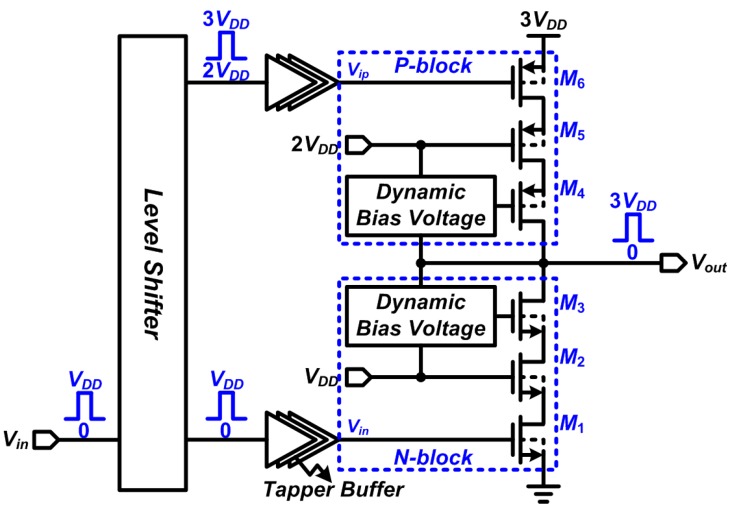
Proposed neural stimulator using the Dynamic Bias Voltage technique.

A current-mode stimulator provides controllable injected current over predefined load variations but with degraded conversion efficiency caused by reduced voltage headroom [14]. A voltage-mode stimulator features high efficiency, but with integration difficulties and relatively expensive fabrication processes for high-voltage compliance implementation. Since the primary goals of our study are to increase conversion efficiency to extend operational lifespan and prostheses miniaturization with high degree of integration, the simplified circuit schematic proposed for our DBS is shown in Figure 3.

**Figure 4:**
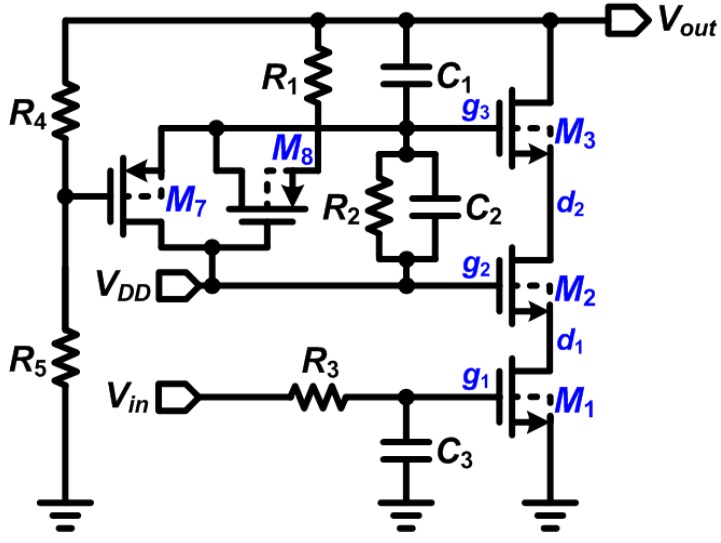
Circuit schematic of the Dynamic Bias Voltage at the stacked discharge transistors *M*_1_–*M*_3_.

The proposed design inherits the dynamic biasing concept demonstrated in a previous study [28] that can render a power supply two to three times higher than its nominal supply voltage level. This process can be realized in a triple-well standard complementary metal-oxide-semiconductor (CMOS) process without requiring of special high-voltage masks. The incorporation of a dynamic biasing affords the system the capability of interfacing with high-voltage supplies using standard low-voltage devices provided by foundry. For standard metal-oxide-semiconductor (MOS) devices, the voltage (or potential) difference between any two terminals in a transistor must not exceed the nominal supply voltage to avoid breakdown. By taking advantage of symmetry, the circuit can be explained using only the discharging part, as shown in Figure 4. The dynamic biasing sub-circuit generates a voltage on *g_3_* to control the gate terminal of transistor *M*_3_. The voltage on *g*_3_ varies with two *V_out_* states.

When *V_out_* is “High” (represented in this study by 3 × nominal transistor voltage), the voltage divided by *R*_4_ and *R*_5_ forces the transistor *M*_7_ to become “OFF”. The transistor *M*_8_ turns “ON” and forms a conduction path via *V_out_*-*g*_3_-*g*_2_. The voltage divided by *R*_1_ and *R*_2_ on the path is properly set for *g*_3_. When *V_out_* is “Low” (represented in this study by nominal transistor voltage), the transistor *M*_7_ becomes “ON” and the *g*_3_ is directly connected to the *g*_2_ with the nominal supply voltage V*_DD_* to turn off transistor *M*_8_. When *V_out_* changes from “Low” to “High”, the transistors *M*_1_, *M*_2_, and *M*_3_ change their states of operation. During *V_out_* = “Low”, all the transistors operate in the triode region and *V_out_* discharges at the ground (0 V or the lowest potential) through *d*_1_ and *d*_2_.

When the *V_in_* changes from V*_DD_* to 0 V, the transistor *M*_1_ starts to enter the cutoff region, where *d*_1_ is first charged to a voltage level of V*_g_*_2_–V_thn_ (V_thn_ represents the threshold voltage of n-channel MOS) followed by the full voltage of V*_g_*_2_ with sub-threshold currents. To prevent transistor *M_1_* from breakdown, V*_g_*_2_ should be set as V*_DD_*. When the *d*_1_ becomes V*_g_*_2_–V_thn_, transistor *M*_2_ then starts to switch off and *d*_2_ starts to become charged. Like those of *M*_1_, *d*_2_ is first charged to a voltage level of V*_g_*_3_–V_thn_ then followed by the full voltage of V*_g_*_3_ with sub-threshold currents. To prevent the transistor *M*_2_ from breakdown, V*_g_*_3_ should not exceed 2 × V*_DD_*. The size of each of the components and transistors should be carefully controlled.

Finally, transistor *M*_3_ enters the saturation region and *V_DS_* becomes *V_DD_* for both the *M*_1_ and *M*_2_. Theoretically, the output voltage of stimulator can be as high as 3 **×***V_DD_*, owing to the stacked P-transistors operating in the triode region. However, the equivalent “ON” resistance contributed by the P-transistors forms a voltage divider of ON resistance *R_ON_* and the output load *R_L_*, which can slightly decrease the output voltage of the stimulator. The formation of these resistances renders *V_DS_* of *M*_3_ to less than *V_DD_* and can benefit the transient operation of the circuit.

When *V_out_* changes from “High” to “Low”, the previous states of transistors *M*_1_, *M*_2_, and *M*_3_ operate in the cutoff region and the output is initially high. Then, transistor *M*_1_ first responds to the input changes and enters the triode region, which discharges *d*_1_. Setting the *d*_2_ at *V_DD_* ensures that *V_GS_* of *M*_2_ does not exceed *V_DD_*. When *d*_1_ discharges to V*_g_*_2_–V_thn_, transistor *M*_2_ enters the cutoff region because of the charged *d*_2_. Similar to the operation of *M*_1_, *d*_2_ is first charged to V*_g_*_3_–V_thn_ and then to the full voltage of V*_g_*_3_ by the subthreshold currents. To prevent transistor *M*_2_ from breakdown, V*_g_*_3_ should not exceed 2 × V*_DD_*. Finally, transistor *M*_3_ enters the cutoff region and *V_DS_* becomes *V_DD_* for both *M*_1_ and *M*_2_.

**Figure 5:**
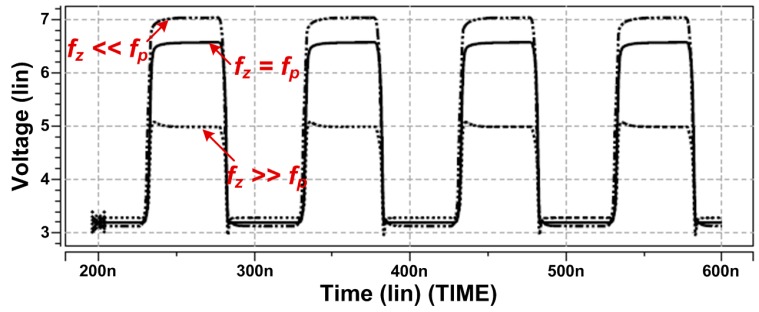
Simulation results of *g*_3_ for *R*_1_–*C*_1_ and *R*_2_–*C*_2_ combinations. The voltage variations in *g*_3_ and the importance of transistor sizing were demonstrated. Overstress failure in *M*_3_ was observed for *g*_3_ exceeding 2 × *V_DD_* with *f_z_* << *f_p_*.

It is worthwhile to note that the *R*_2_–*C*_2_ combination forms a zero *f_z_* and *R*_1_–*C*_1_ combination forms a pole *f_p_*. They should be sized carefully when designing the stimulators. The voltage variations in *g*_3_ through HSPICE-based simulations demonstrate the importance of transistor sizing. As shown in Figure 5, when *g*_3_ exceeds 2 × *V_DD_* if *f_z_* << *f_p_*, the high voltage will cause overstress failures in *M*_3_. The relationships between the output and gate voltages must be controlled precisely for increased system reliability.

The residual charges of the system can be cancelled (charge balance) by shorting the stimulator output to a negative supply, whose absolute voltage level is the same as the positive rail. The involved MOS switch is “ON” during the non-stimulation phase. The occurrence of action potentials or extra stimulus damage stems from a stimulation current which exceeds a threshold. Merrill *et al.* detailed in their paper a strength-duration curve for the stimulation, where minimum current required for stimulation, defined as Rheobase current, accompanies with a long stimulus pulse [29]. The Rheobase current is a function of membrane time constant. Simmons *et al.* have shown in their paper that a concrete approximation of the Rheobase current is 6.7 μA [30]. As a result, we set the absolute value of activation threshold as 5 μA and used this numerical value as constant charge cancellation current for charge balance. The path of −5-μA constant current was switched on or off according to a comparator output (the gate terminal of MOS discharge switch was connected to the comparator output). The comparator is essentially an open-loop dual-supply differential amplifier whose inputs were connected to the ground and *V_out_*. This forms a closed-loop charge balance.

**Figure 6:**
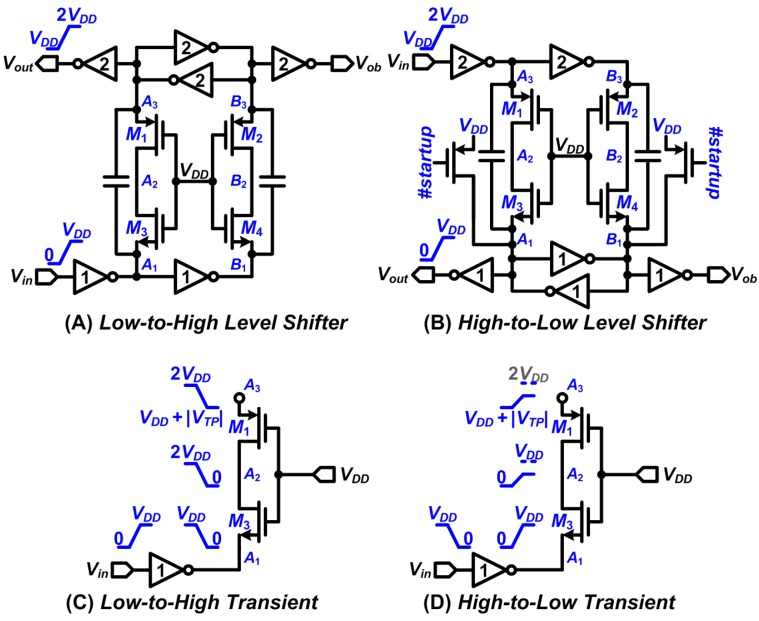
Proposed level shifters and their transient response analysis.

The level shifters were also demonstrated to play key roles in the DBS stimulator. The shifters output two sets of synchronizing signals to simultaneously drive the *P-block* and *N-block*. Two types of circuit were designed to realize the level shifters, which were the *Low-to-High* and *High-to-Low* circuits, shown in Figure 6A,B. The inverters marked with 1 and 2 output voltages ranging from 0 V to *V_DD_* and *V_DD_* to 2*V_DD_*, respectively. The operations of the level shifters are symmetrical, and consequently they can be explained with one scheme. The schematic of the Low-to-High level shifter is shown in Figure 6.

The signaling of *A*_1_ and *A*_3_ are in phase. Assuming the initial levels of *A*_1_ and *A*_3_ are *V_DD_* and 2*V_DD_*, respectively. The resulting node voltage changes in response to *V_in_* transients is illustrated in Figure 6C. It should be noted that the transistor *M*_3_ of the *Low-to-High* level shifter becomes “Off” when the voltage of node *A*_1_ reaches *V_DD_*–*V_TN_*. Thus, prohibiting *A*_1_ from exceeding *V_DD_* while *A*_3_ is being pulled up. In the same concept, the transistor *M*_1_ becomes “Off” when the voltage of node *A*_3_ is at *V_DD_* + |*V_TP_*|. This reduction in voltage prohibits *A*_3_ from being lower than *V_DD_* while *A*_1_ is being pulled down. This circuit design prevents device breakdown and circuit failure. Finally, the latches formed by the cross-coupled inverters can further stabilize the logic levels of *A*_3_ and *B*_3_.

A special feature for the two level shifters is also proposed for the start-up circuit, which is depicted in the *High-to-Low* level shifter schematic (also exists in the *Low-to-High* counterpart with exact symmetrical circuit configuration and controlled out of phase and inverted signaling signals). Taking the *High-to-Low* level shifter as an example, there will be a propagation delay between the *A*_1_ and *B*_1_ and *A*_3_ and *B*_3_ pairs. Due to the earlier signaling of *A*_3_ and *B*_3_ than that of *A*_1_ and *B*_1_, we added the transistors gated by the number of startups (*#startup*) signal to prevent pre-charge voltages of the capacitors in becoming higher than *V_DD_* at their initial states, and thereby preventing transistor breakdown that is caused by device overstress failure.

### 2.3. The DBS Platform

**Figure 7:**
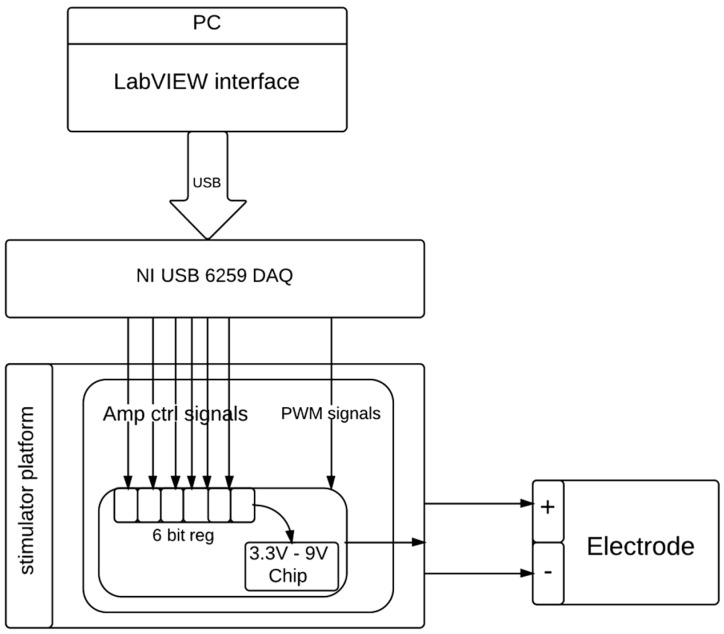
Detailed diagram of device control and the proposed DBS stimulation system.

The proposed system is a programmable electrical stimulator to perform DBS using one independent output channel that supplies the biphasic and constant amplitude to output voltage. The DBS hardware was controlled by a virtual instrument (control program) developed in LabVIEW^®^ 8.6 that communicates with the NI USB-6259 instrument. Figure 7 shows the schematic diagram of the developed interface circuit applied for DBS. In order to prevent excess charge accumulation on the brain tissue, DBS stimulators commonly used biphasic voltage. The variable parameters for stimulation, including frequency, positive pulse width, negative pulse width, and amplitude, were transmitted and stored in the corresponding registers via the address channel and data channel. The proposed DBS system consisted of 63 distinct maximum stimulating voltages where the minimum available voltage span is 90 mV. Four different voltages: 3.3, 5, 7, and 9 V were used for the regulation verification in our animal experiments. The stimulating pulse frequency and width were set using the PWM scheme and the distinct stimulating amplitudes were set using a 6-bit register for each stimulating voltage level. The register set “111111” corresponds to a mode of the highest power supply and stimulator output. In this study, the controlled pulse width and frequency were fixed at 0.3 ms and 3 Hz, respectively.

### 2.4. In Vivo Thalamic Stimuli and in Vivo Impedance Measurement

Five male Wistar rats weighing from 250 to 300 g (BioLASCO Taiwan Corp., Taipei, Taiwan) were used in the electrophysiological experiment. All procedures for the animal experiments were performed in accordance with the Guidelines for Care and Use of Experimental Animals outlined by the Laboratory Animal Center at National Yang Ming University and National Yang Ming University.

Under isoflurane anesthesia (3.5% induction; 1.5% maintenance; Hospira, Lake Forest, IL, USA), the rats were placed on a standard stereotaxic apparatus (Model 900, David Kopf Instruments, Tujunga, CA, USA). Two craniotomies were performed and epidural cortical electrodes were secured into the skull over the bilateral primary somatosensory cortex of the forepaw (S1FL) at 0.5 mm anterior and bi-lateral to Bregma for ECoG recordings. A stainless steel screw positioned at 2-mm posterior and 1-mm lateral to lambda was used as the reference electrode. The two epidural cortical electrodes and the reference screw were permanently cemented to the skull using dental acrylic (Type 1 Class 1, Hygenic Corp., Akron, OH, USA). For chronic thalamic stimulation, another craniotomy was performed for the implantation of the twisted bipolar platinum–iridium electrodes. The electrodes were of 75 μm in bare diameter, 155 μm in insulated diameter, with an overall 10 mm electrode length, and were exposed only at the tip transection at approximately 0.08 mm apart between the electrode tips. The electrodes were placed at the thalamic ventral posteriolateral (VPL) nuclei, which were at 3 mm posterior and 3 mm right relative to Bregma, and 6 mm ventral from the cortical surface, respectively [31]. The twisted bipolar electrodes acted as the anode and cathode for local stimulation and were secured onto the skull using dental acrylic and covered with a small amount of 2% agar. Rectal temperature was measured with a thermocouple and maintained at 37 ± 0.5 °C through a feedback-controlled blanket system (Harvard Apparatus, Holliston, MA, USA).

We have demonstrated the capability of using the standard 3.3-V triple-well CMOS device to accommodate a stimulation output as high as over 9 V. For thalamic stimuli, biphasic constant voltages at 3.3, 5, 7.5, and 9 V pulses, with widths of 0.3 ms at a frequency of 1 Hz, were administered using the DBS stimulator prototype to each of the anesthetized rats. Meanwhile, bilateral somatosensory evoked potentials (SSEPs) were filtered on pre-amp between 0.3 to 250 Hz and sampled at 1 kHz. Data acquisition was performed using the laboratory-designed multi-channel data acquisition system [32].

The *in vivo* electrode-electrolyte interface impedance was measured through the twisted bipolar electrodes by an impedance spectrometer (LCR4235, Wayne Kerr Electronics Ltd., West Sussex, UK) with a sinusoidal voltage of 20 mV, <150 nA, at 1 kHz. The *in vivo* impedance measurement parameters were based on the standard methods adopted from other previous studies [33,34]. After implantation, the twisted bipolar electrodes’ *in vivo* electrode-electrolyte interface impedance, at 1 kHz, was measured at 0.172 ± 0.023 MΩ (mean ± S.D., *n* = 5).

### 2.5. Electrophysiological Data Analysis

The SSEPs were analyzed offline using MATLAB (MATLAB R12, Mathworks Inc., Natick, MA, USA) to evaluate the evoked responses induced by thalamic electrical stimuli. The evoked SSEP amplitudes of the individual sweeps were averaged over 50 sweeps to generate an average evoked SSEP. The averaged evoked SSEP was then summed to obtain an absolute value of the amplitudes for the evoked response during the 180-ms post-stimulus period, denoted as ΣSSEP. The changes in ΣSSEP were used to evaluate the stabilities of the evoked responses induced by the thalamic stimuli over long periods. Furthermore, the coefficient of determination (*R*^2^) of the linear curve fit was statistically evaluated from the relationship between the ΣSSEP and each of the stimulus intensities that include 3.3, 5, 7.5 and 9 V. The *R*^2^ value of above 0.8 was set as the indication of a statistically good fit. The resulting data, with mean values, standard deviation (Mean ± S.D.), *in vivo* impedance, and ΣSSEP are presented in the following section.

## 3. Results and Discussion

### 3.1. Chip Testing and Characterization

The proposed voltage-controlled stimulator has been fabricated by a standard 0.18-μm triple-well CMOS process. It was implemented based on the simple inverting circuit structure that uses the standard input/output (I/O) MOS devices provided by the foundry. Since the impedances of the twisted bipolar electrodes’ *in vivo* electrode-electrolyte interface have been measured, we designed our stimulator, with appropriate transistor sizing, to cover an interface for ranges from 0.5 MΩ (its optimal performance) to 0.1 MΩ ( required for clinical DBS applications) [35].

**Figure 8:**
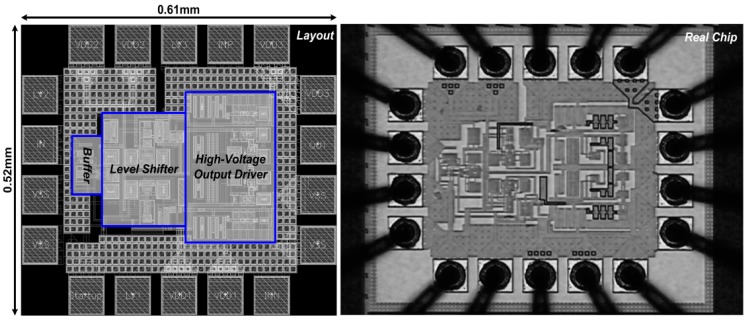
Photomicrograph of fabricated stimulator chip. The chip design was implemented in 0.18-μm standard triple-well CMOS process and measured at 0.61 × 0.52 mm^2^.

**Figure 9:**
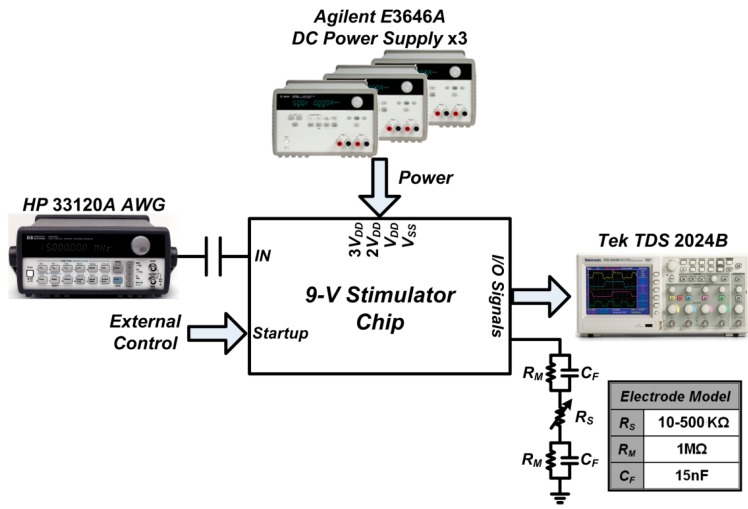
Neural stimulator measurement setup.

Figure 8 shows the die photo of the fabricated chip that supports for up to 9 V of stimulation voltage. The voltage conversion efficiency (VCE) was measured as the output voltage, with a dummy load representing the practical conditions, divided by the power supply and was expressed as a percentage. The measurement setup of the proposed stimulator function is illustrated in Figure 9. The Startup function served as a fail-safe soft-start mechanism. It was designed to set the initial voltages of the internal stimulator nodes after power-on to prevent overstress failure and transistor breakdown. A function/agilent waveform generator (33120A, Hewlett Packard, Palo Alto, CA, USA) was used to control the stimulus pulse parameters. 

**Figure 10:**
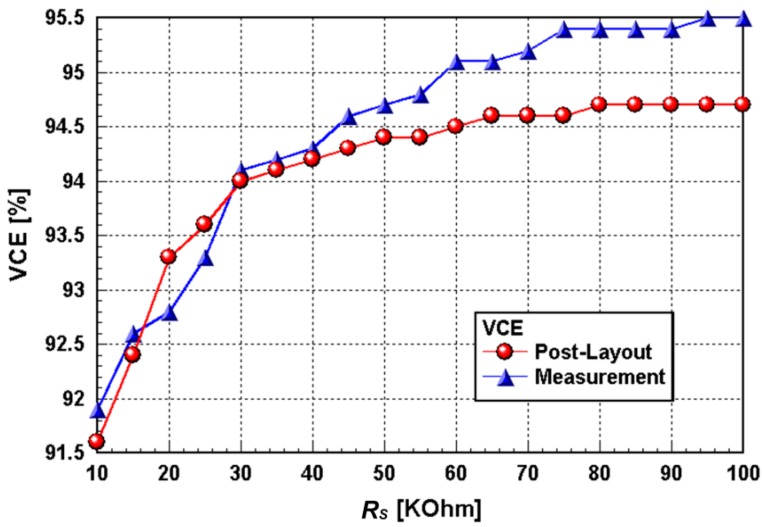
Simulated and measured voltage conversion efficiencies (VCEs) *vs.* a dummy load (*R_S_*).

**Figure 11:**
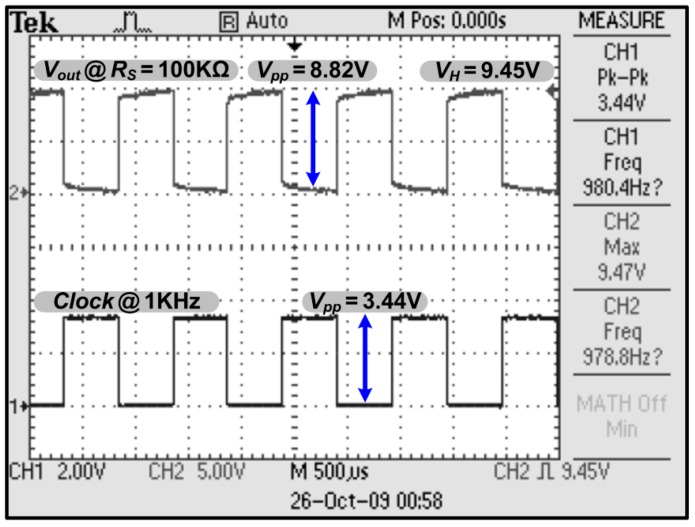
Oscilloscope traces of prototype stimulator working at 9 V DC with an *R_S_* of 100 kΩ.

The resistor *R_M_* and capacitor *C_F_* are precise components used to emulate the practical conditions of the interface. The interface with the target of interest can be represented by the half-cell potential consisting of an R//C-R network [36]. The chip characterization experiments were performed for understanding the real device output performance and efficiency. The VCE measurement exhibited the highest efficiency at about 95% under a dummy load (*R_S_*) of 100 kΩ (Figure 10). The measurement and simulation results of the chip were in good agreement with each other. The VCE measurements showed better device efficiencies than what was demonstrated in the simulations. This increase in efficiency can be attributed to the overestimation parasitic effect of the involved transistors. The fabricated stimulators have almost identical circuit performances. Figure 11 shows the oscilloscope traces of the prototype stimulator working at 9 V DC with a *R_S_* of 100 kΩ. The input signal used to switch the stimulator is at 1-kHz clock speed. The highest output potential of the stimulator is almost triple that of the input signal.

Despite a number of physical failure mechanisms, modern CMOS integrated circuit (IC) was designed to have up to 20 to 40 year reliability. This is because CMOS devices are created for the purpose of being placed into consumer devices and the length of their service time should be as long as possible. Our circuit design underwent rigorous design processes, implementation, and verification to ensure the reliability of the final product. All of the proof-of-concept stimulators underwent a 24-h field trial without interruption in our lab and have exhibited no efficiency degradation. This stability result demonstrated the reliability of our circuit design. The implantable DBS device contains a small battery that produces the electrical pulses required for stimulation. The typical battery life is expected to be approximately five years. However, this estimated battery life may vary depending on the individual settings and hours of use per day [37]. Despite the difficulty of confirming the service life of our design experimentally, the current results suggest that it is promising for long-term reliability. Compared to the recent works in other studies [9,10,11,12,13], our study demonstrated a truly high-voltage-mask-free neural stimulation chip design with low cost and ease of integration. Table 1 shows a comparison of our prototype stimulator against other devices demonstrated in the literatures.

**Table 1:** Comparison of neural stimulators.

Reference	[9]	[10]	[11]	[12]	[13]	*This work*
Process	0.35-μm HV CMOS	0.35-μm HV CMOS	0.8-μm HV CMOS	0.8-μm HV CMOS	0.35-μm HV CMOS	0.18-μm Standard CMOS
Power Supply	11.7 V	11.7 V	±9 V	10 V	20 V	9.9 V
Output Load	60 kΩ	>10 kΩ	148.2 kΩ	9.125 kΩ	1-10 kΩ	10-500 kΩ

### 3.2. In Vivo Stimuli in Rat Thalamus Using the Prototype DBS System

**Figure 12:**
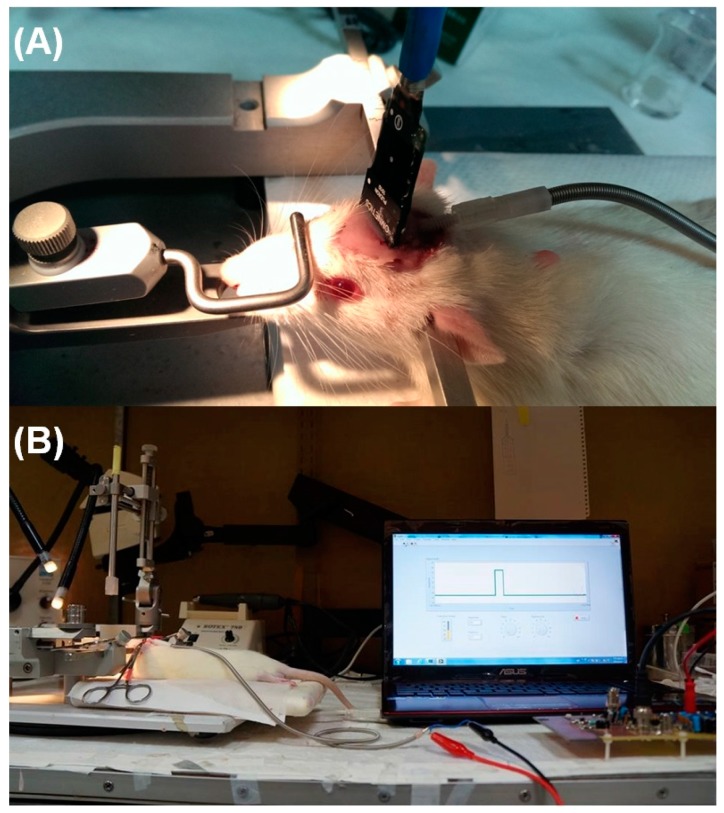
(**A**) Experimental setup for in vivo stimulation in the rat thalamic VPL area with a two-channel platinum-iridium electrode and screw electrodes with Omnetics headstage-preamplifiers for ECoG recording; (**B**) User interface of the virtual instrument developed in LabVIEW^®^ for controlling the prototype DBS hardware system.

*In vivo* animal experiments were performed with our prototype DBS system to demonstrate the feasibility of our DBS system. Figure 12 shows a photograph of the experimental setup. In this study, the prototype DBS system was set with biphasic voltage-controlled conditions. The voltage amplitudes were set at 3.3, 5, 7.5, and 9 V, with pulse durations and frequencies of 0.3 ms and 3 Hz, respectively. Figure 12A shows the two-channel platinum-iridium electrode that was inserted into the VPL thalamus for DBS. The evoked responses induced by the electric stimulation with the DBS system were measured in the S1FL cortex in the brain. Screw electrodes were inserted through the skull of the rat, and ECoG in the S1FL cortex corresponding to the DBS was observed. The LabVIEW^®^ program that was used for performing the DBS experiments was shown in Figure 12B. This graphical user interface (GUI) enabled all stimulus parameters to be adjusted individually for every DBS task, for example, amplitude, pulse width, and frequency can be changed to verify the evoked ECoG response. 

**Figure 13:**
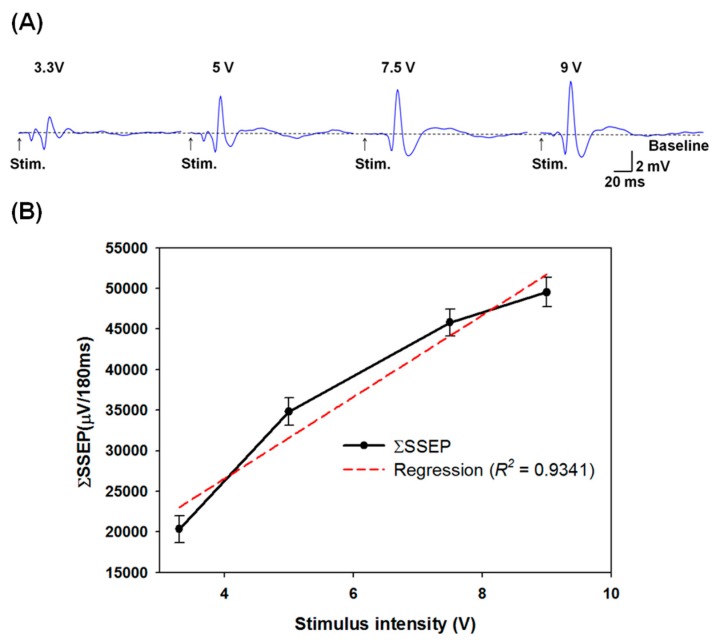
(**A**) The SSEP morphologies obtained from ECoG with different stimulus intensities (3.3, 5, 7.5, and 9 V). The responses were averaged across 50 repetitions. The arrowheads indicate the stimulating moments for each stimulus intensity; (**B**) The mean averaged evoked SSEP (ΣSSEP) averaged from the electrocorticography (ECoG) recordings were obtained from five rats with different stimulus intensities. The linear curve fit shows a strong correlation (*R*^2^ = 0. 9341) between the ΣSSEP and the stimulus intensity.

### 3.3. Grading Thalamic Stimuli Induced Somatosensory Evoked Potentials

An example of thalamic stimulation induced SSEP is shown in Figure 13A. The increase in thalamic stimuli intensities was shown to result in increased SSEP magnitudes. The averaged ΣSSEPs for 3.3, 5, 7.5, and 9 V thalamic stimuli were 20.332 ± 1.297, 34.801 ± 1.379, 45.829 ± 1.294 and 49.530 ± 1.311 mV/180 ms, respectively. The relationship between the ΣSSEP results and stimulus intensities was also examined. The black line in Figure 13B represents the linear curve fit (*R*^2^ = 0.9341). The results suggest that the ΣSSEP results and the stimulus intensities exhibited a significant linear relationship, demonstrating that our highly integrated low-cost and energy-efficient DBS system is promising for the effective treatment of neurodegenerative diseases [38,39,40,41,42].

### 3.4. In Vivo Electrochemical Characterization for Electrode-Electrolyte Interface and the Evaluation of its Effect on the Stimulator

In addition to the dummy load that was used to emulate an actual state of electrode-electrolyte interface, further testing with a real typical load that included electrode impedance, wiring capacitance, parasitic impedance, and tissue can be beneficial for evaluating the electrode-electrolyte interface and the functionality of the fabricated stimulator chip. Several test signals with ultra-low output impedance were generated and connected to the working electrode, and the test signal and its resulting feedback were further analyzed. We performed the electrochemical characterization of the implanted electrode *in vivo* using electrochemical impedance spectroscopy (EIS) and cyclic voltammetry (CV) experiments. EIS and CV measurements were performed on a VersaSTAT 4 (AMETEK, Advanced Measurement Technology, Oak Ridge, TN, USA). 

**Figure 14:**
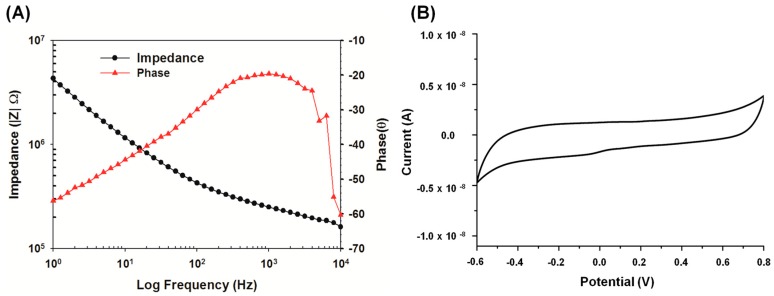
Electrochemical characterization of electrode-electrolyte interface after implantation (**A**) EIS measurement and (**B**) CV curve of implanted electrode with a scan rate of 50 mV/s.

EIS measurements were performed on a Gamry potentiostat from 1 Hz to 20 kHz with a 20-mV alternating current (AC) amplitude. CV measurements were performed in a potential range between the water electrolysis window of −0.6 V to 0.8 V at a scan rate of 50 mV/s [43]. The stimulating electrode for EIS and CV analysis were shown in Figure 14. EIS provided *in vivo* impedance measurement at 1 kHz was about 250 kΩ as shown in Figure 14A. The impedance value corresponds to a slew time (the average time of rising and falling of the stimulator output) of approximately 5 ns. By taking the average slew voltage of the output from the average slew time, we concluded that the stimulator chip has a measured slew rate of about 1.7 V/ns. 

The CSC is calculated from the time integral of the cathodic and anodic areas in a slow scan rate of 50 mV/s from *in vivo* CV measurement and then normalized with respect to geometric surface area of our implanted electrode [44]. In this study, our platinum-iridium electrodes bring the charge storage capacity to 860 μC/cm^2^ *in vivo*. Cyclic voltammetry analysis also showed stable CV traces of the implanted electrode without anodic and cathodic peak currents, that there was no reduction/oxidation reaction occurred in the electrode-electrolyte interface, as shown in Figure 14B.

### 3.5. Advantages and Discussions of the Proposed System 

The developed platform provides clinicians with easier understanding of the meditation of DBS and its effect on brain activity. With simultaneous ECoG measurements and recordings, one can determine the relevance between intracortical stimulation and neural cortex activity. This data can be utilized by the clinicians for performing noninvasive studies such as electroencephalography (EEG) analysis. Currently, general DBS devices are rarely equipped with concurrent recording circuits to record brain activity, the prototype device demonstrated herein with integrated ECoG measurement can become a promising tool for understanding related neurological disorders. 

## 4. Conclusions

In recent years, we have witnessed a dramatic increase in the number of electronic devices used for medical applications such as neural recording [34,45,46,47,48,49]. DBS has been an important surgical procedure in which a device called a neurostimulator delivers tiny electrical signals to brain areas that are related to movement control. Symptom improvement in essential tremors such as those caused by Parkinson’s disease has been reported and well documented. This study presented a new high-efficiency and low-cost neural stimulator design with the potential to be utilized as a new-generation implantable therapeutic and prosthetic device. To the best of our knowledge, the proposed DBS is the first miniaturized electrical stimulation chip implemented with a general purpose process for electrical stimuli control with widely different intensities. The prototypes were fabricated with standard semiconductor technology and their implementation was demonstrated within *in vivo* measurements with a custom platform. For the demonstration of *in vivo* DBS, our electrophysiological results have shown that the evoked SSEPs and the stimulus intensities from our prototype system were shown with significant linear correlation. The system can be controlled by adjusting the stimulus voltage required for the activation of postoperative neural response. However, it is prone to interface impedance variations [23,50]. In order to ensure optimal system performance, the output of the stimulator should be equipped with a high-voltage supply. The system can be further improved with advanced electrode designs, and thus efforts are still being made for balancing between optimal electrode and stimulator designs. Our team has been developing several novel DBS electrodes with improved performance [51,52]. The integration of the prototype DBS stimulator chip with the miniaturized electrodes, other front end sensors, and required signal processors for applications in medical therapies with extreme space-constrained may become possible in the near future.
